# The MTA family proteins as novel histone H3 binding proteins

**DOI:** 10.1186/2045-3701-3-1

**Published:** 2013-01-03

**Authors:** Meng Wu, Lina Wang, Qian Li, Jiwen Li, Jun Qin, Jiemin Wong

**Affiliations:** 1 Shanghai Key Laboratory of Regulatory Biology, Institute of Biomedical Sciences and School of Life Sciences, East China Normal University, Shanghai, 200241, China; 2 Center for Molecular Discovery, Verna and Marrs McLean Department of Biochemistry and Molecular Biology, Baylor College of Medicine, Houston, TX, 77030, USA; 3 Institute of Biomedical Sciences and School of Life Sciences, East China Normal University, 500 Dongchuan Road, Shanghai, 200241, China

**Keywords:** MTA1/2/3, NURD complex, Histone H3 tail, Histone deacetylation, Chromatin

## Abstract

**Background:**

The nucleosome remodeling and histone deacetylase complex (Mi2/NRD/NuRD/NURD) has a broad role in regulation of transcription, DNA repair and cell cycle. Previous studies have revealed a specific interaction between NURD and histone H3N-terminal tail in vitro that is not observed for another HDAC1/2-containing complex, Sin3A. However, the subunit(s) responsible for specific binding of H3 by NURD has not been defined.

**Results:**

In this study, we show among several class I HDAC-containing corepressor complexes only NURD exhibits a substantial H3 tail-binding activity in vitro. We present the evidence that the MTA family proteins within the NURD complex interact directly with H3 tail. Extensive in vitro binding assays mapped the H3 tail-binding domain to the C-terminal region of MTA1 and MTA2. Significantly, although the MTA1 and MTA2 mutant proteins with deletion of the C-terminal H3 tail binding domain were assembled into the endogenous NURD complex when expressed in mammalian cells, the resulting NURD complexes were deficient in binding H3 tail in vitro, indicating that the MTA family proteins are required for the observed specific binding of H3 tail peptide by NURD in vitro. However, chromatin fractionation experiments show that the NURD complexes with impaired MTA1/2-H3 tail binding activity remained to be associated with chromatin in cells.

**Conclusions:**

Together our study reveals a novel histone H3-binding activity for the MTA family proteins and provides evidence that the MTA family proteins mediate the in vitro specific binding of H3 tail peptide by NURD complex. However, multiple mechanisms are likely to contribute to the chromatin association of NURD complex in cells. Our finding also raises the possibility that the MTA family proteins may exert their diverse biological functions at least in part through their direct interaction with H3 tail.

## Background

Histone lysine acetylation plays a central role in the epigenetic regulation of gene expression [1-3]. The levels of histone acetylation in eukaryotic cells are governed by the opposing activities of histone acetyltransferases(HATs) and histone deacetylases (HDACs) [3]. The mammalian genome encodes a significant number of HDACs, among them are the abundantly and ubiquitously expressed class I HDACs that include HDAC1, HDAC2 and HDAC3 [3]. The class I HDACs are mainly found in stable multi-subunit complexes with co-repressor proteins like Sin3A [4,5], CoREST [6,7], CHD3/CHD4(Mi2-α/β) [8,9] and NCoR/SMRT [10,11]. The NURD complexes, also widely known as Mi-2/ NRD/NuRD complexes [8,9,12,13], have been independently purified from various sources. Although the NURD complexes purified by different laboratories vary slightly in compositions, it is generally agreed that the NURD complex contains either CHD3 (also known as Mi2-α) or CHD4 (also known as Mi2-β), HDAC1 and HDAC2, RbAp46 (also known as Rbbp7) and RbAp48 (also known as Rbbp4), MTA1/2/3, MBD3, and p66α and p66β subunits [14]. The presence of CHD3 or CHD4 subunit renders the NURD complex the unique ATP-dependent chromatin remodeling activity that is coupled with HDAC activity [8,9,12,13]. Of note, the MTA family proteins MTA1 (metastasis-associated gene 1), MTA2 and MTA3 form exclusive NURD complex. The NURD complex is highly conserved in evolution and has been shown to have important and broad roles in transcription, DNA repair, cell cycle and genome stability [15,16].

In principle, various HDAC complexes can play a role in histone deacetylation either through a recruitment mechanism via interaction with sequence-specific transcription factors or chromatin associated proteins and/or direct interaction with chromatin [17,18]. In this regard, previous studies demonstrated that the NURD, but not the similar Sin3A complex that share with NURD the core HDAC1/2 and RbAp46/48 subcomplex, interacts with the histone H3N-terminal tail in vitro [19,20]. In fact, the NURD complex appeared to be the major H3 tail peptide-proteins in the HeLa nuclear extracts. Furthermore, the NURD-H3 interaction is diminished when H3K4 site is methylated [19,20]. While this outstanding interaction with H3 tail implies a critical role of NURD complex in control of histone acetylation, surprisingly it is not yet clear what protein subunit(s) within the NURD complex is responsible for this specific interaction with H3 tail. As the core HDAC1/2 and RbAp46/48 subcomplex is common between Sin3A and NURD, efforts have been focused on the proteins unique to the NURD complex including CHD3/4 and p66α and p66β subunits. The CHD3 and CHD4 are attractive candidates since both proteins contain two PHD domains [21]. PHD domains from various proteins have emerged as methylated and unmodified histone binding modules [22]. Musselman et al. reported the interaction of the second PHD domain in CHD4 with H3 tail peptide and more recently the bivalent recognition of two H3 tails in a nucleosome by CHD4 tandem PHD domains [23,24]. However, it is unclear if CHD4 and/or CHD3 are responsible for the observed H3-binding activity of the NURD complex. Another study reported the interaction of p66α and p66β subunits with histones [25]. However, the p66α and p66β subunits were found to interact with not only H3 but also H2A, H2B and H4, a result inconsistent with the observed specific interaction of NURD with H3 but not other histone N-terminal tails [19,20]. Thus, exactly how the NURD complex recognizes specifically H3 tail remains to be determined.

In this study, we have investigated the H3 tail binding specificity of individual subunits of NURD complex and present the evidence that the MTA family proteins are novel histone H3 binding proteins. MTA1, the founding member of the MTA family proteins that include MTA1, MTA2 and MTA3, was initially identified as a metastasis-associated tumor gene [26,27]. Previous studies indicated that the MTA proteins form exclusive alternative NURD complexes and have broad function in transcriptional regulation [28,29]. Furthermore, previous study indicated that the MTA1 can also form a core complex containing other subunits of NURD complex except CHD3 or CHD4 [30]. We mapped the H3 binding activity to the C-terminus of MTA1 and MTA2. We present the evidence that the MTA family proteins within the NURD complex interact directly with H3 tail and this interaction most likely accounts for the observed specific binding of H3 tail peptide by NURD in vitro. However, chromatin fractionation experiments show that the NURD complexes with impaired MTA1/2-H3 tail binding activity remained to be associated with chromatin in cells. Our study provides new insight for binding of chromatin by NURD and uncovers a novel histone-binding activity for MTA family proteins.

## Results

### Unique and robust binding of H3 tail peptide by NURD but not other class I HDAC complexes

**Figure 1:**
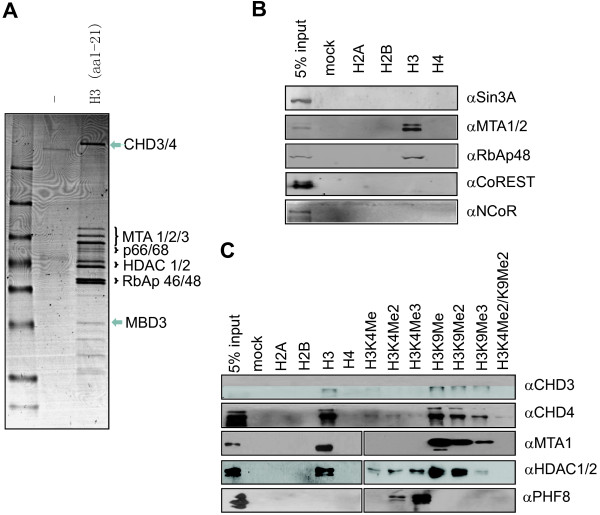
**Unique binding of H3 tail peptide by NURD but not other class I HDAC complexes. A**. Coomassie blue staining revealed the H3 tail peptide-binding proteins isolated from HeLa nuclear extracts. The identities of the protein bands were determined by mass spectrometry. ‘-‘, the beads only control. **B**. Among several class I HDAC complexes only NURD exhibited a substantial H3 tail-binding activity in vitro. The HeLa nuclear extracts were incubated with various immobilized histone tails in vitro and the binding of various class I HDAC complexes was examined by western blot analysis using antibodies against complex-specific subunits. Sin3A, the unique subunit of the Sin3A/HDAC1/2 complex; CoREST, the unique subunit of CoREST complex; MTA1/2, the unique subunits of NURD; NCoR, the unique subunit of NCoR/SMRT/HDAC3 complex. **C**. The binding specificity of NURD complexes toward histone H3 tail peptides. The HeLa nuclear extracts were incubated with the immobilized histone tail peptides as indicated and the binding of NURD complexes was examined by subsequent western blot analysis using antibodies against CHD3, CHD4, MTA1 and HDAC1. PHF8 served as a positive control for H3K4me2/3-binding proteins.

To identify proteins that potentially read the unmodified histone H3N-terminal tail, we synthesized a H3 1–21 aa peptide with a C-terminal biotin moiety. This peptide was first immobilized onto the streptavidin agarose beads and then incubated with proteins in HeLa nuclear extracts. After extensive washes, the proteins that bound to the beads were boiled in SDS-loading buffer, resolved with a 4-20% SDS-PAGE gel and visualized by coomassie blue staining (Figure 1A). The resulting H3 peptide-binding protein bands were excised, digested with trypsin in-gel, and subjected to protein identification by mass spectrometry. As shown in Figure 1A, all the major protein bands were identified as components of the mammalian NURD complex [8,9,12,13], including the CHD3/4, MTA1/2/3, p66α/β, HDAC1/2, RbAp46/48 and MDB3. In fact, the protein band pattern in Figure 1A is essentially identical to the NURD protein complex extensively purified by conventional and immunoaffinity chromatography as reported previously [8,9]. This result is in well agreement with previous studies [19,20], and clearly demonstrated that the NURD complex is the major and abundant histone H3N-terminal tail-binding proteins at least in the HeLa nuclear extracts.

In addition to the NURD complexes, HeLa nuclear extracts contain also abundantly the HDAC1/2-containing Sin3A and CoREST complexes. However, in our mass spectrometry analysis we did not identify any proteins that are unique to Sin3A, a result consistent with previous studies [19,20]. Furthermore, we also failed to identify unique components of the CoREST complexes, indicating both Sin3A and CoREST corepressor complexes did not bind the H3 peptide under our experimental conditions. To test this further, we carried out pulldown assays with biotinylated H2A, H2B, H3 and H4N-terminal peptides and HeLa nuclear extracts, and the resulting binding proteins were analyzed by western blot using antibody against Sin3A and CoREST. Consistent with our mass data, western blot analysis using an antibody that is specific for both MTA1 and MTA2 detected the significant enrichment of MTA1 and MTA2 in the H3 but not other histone peptides-bound fractions. As expected, western blot analysis also detected the enrichment of RbAp48 by H3 but not other peptides. However, Sin3A and CoREST were detected only in the input but not in the H3 bound fraction (Figure 1B). Similarly, we found that the NCoR/SMRT corepressor complexes, which contain HDAC3 but not HDAC1/2, were present in the input but not in the bound fraction. Together these data extend previous observation [19,20] to that the NURD but not other class I HDAC complexes exhibits a unique and robust H3 tail-binding activity.

Previous studies indicated that H3K4 methylation substantially inhibited the H3-binding activity of NURD [19,20]. We confirmed that H3K4 mono-, di-and tri-methylation all inhibited the binding of NURD to H3 peptide (Figure 1C). As a control, PHF8 was found to bind preferentially the K4me3 peptide (Figure 1C) [31,32]. Interestingly, we observed that while K9me1 and K9me2 did not obviously affect the binding of NURD, K9me3 appeared to inhibit the binding.

### Within the NURD complexes the MTA proteins directly bind H3 tail peptide

**Figure 2:**
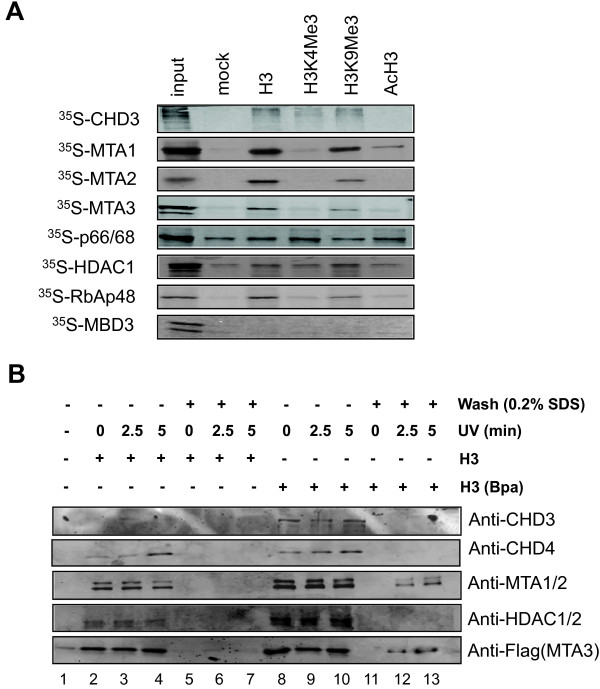
**The MTA family subunits bind directly the H3 tail peptide. A**. the H3 tail peptide-binding specificity of individual subunits of NURD complex. Each subunit of NURD complex was synthesized and ^35^S-met labeled via in vitro coupled transcription and translation. The proteins were then subjected to in vitro pulldown with immobilized H3 tail peptides as indicated. The binding was visualized by autoradiography. Note that the MTA1 family proteins, CHD3 and RbAp48 bound H3 tail in a H3K4me3-sensitive manner, whereas p66/68 and HDAC1 were not. **B**. UV-induced cross-linking demonstrated a direct association of MTA family proteins with H3 tail peptide. The H3(Bpa) peptide contained at K9 position a Bpa moiety that mediated a UV-induced cross-linking with associated protein(s) in close proximity. Note that once cross-linked to the H3(Bpa) peptide, the associated protein(s) became resistant to wash with 0.2% SDS buffer.

**Figure 3:**
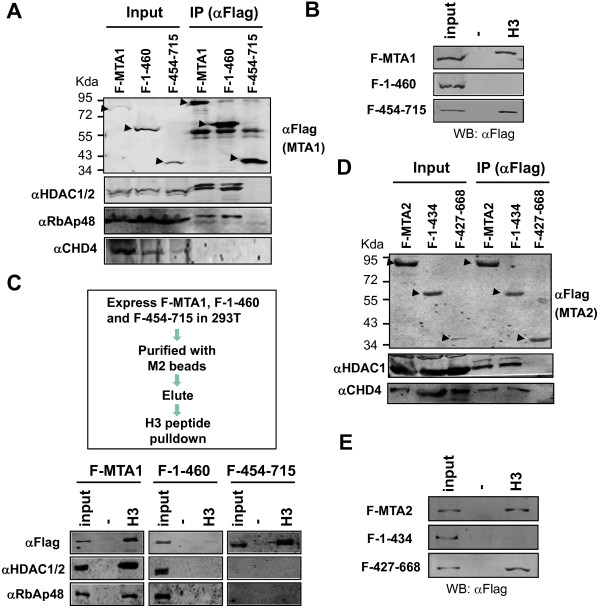
**The H3BD of MTA1 and MTA2 mediates the in vitro binding of H3 tail peptide by NURD. A**. Mammalian expressed MTA1(1–460) but not MTA1(454–715) was incorporated into the core HDAC1/2 and RbAp46/48 containing complex. The MTA1 and its N- and C-terminal regions were expressed in 293T cells and assayed for association with other subunits of NURD complex by IP-western analysis. Note that the N-terminal but not the C-terminal region of MTA1 associated with HDAC1/2 and RbAp48 and that no association with CHD4 was detected for MTA1. Input, 10%. **B**. The MTA1 C-terminal region but not the N-terminal region that was incorporated into the HDAC1/2-RbAp46/48 core complex bound the H3 tail peptide in in vitro pulldown assay. The samples in **A** were subjected to in vitro pulldown assay. Note that no binding was detected for Flag-MTA1(1–460), whereas Flag-MTA1(454–715) bound the H3 tail peptide as efficient as Flag-MTA1. Input, 10%. **C**. The C-terminal region of MTA1 binds H3 tail peptide independent of HDAC1/2 and RbAp48. The 293T-expressed, Flag-tagged MTA1, MTA1(1–460) and MTA1(454–715) were purified as illustrated in the top panel and then subjected to in vitro pulldown assay for binding of H3 tail peptide. **D**. Mammalian expressed MTA2(1–434) but not MTA2(427–668) was incorporated into the endogenous NURD complex. The MTA2 and its N- and C-terminal regions were expressed in 293T cells and assayed for association with other subunits of NURD complex by IP-western analysis. Note that the N-terminal but not the C-terminal region of MTA2 associated with HDAC1/2 and CHD4. Input, 10%. **E**. The C-terminal region but not the N-terminal region that was incorporated into the endogenous NURD complex bound the H3 tail peptide in in vitro pulldown assay. The samples in **D** were subjected to in vitro pulldown assay. Input, 10%.

To identify the protein(s) within the NURD complexes that directly binds the H3 tail peptide, we first expressed and labeled each individual NURD subunit with ^35^S-methionine by in vitro coupled transcription and translation reactions. These proteins were then tested for binding of H3 and H3K4me3, H3K9me3 and acetylated H3 peptides by in vitro pulldown assay. Interestingly, we found that all subunits except MBD3 were able to bind the H3 peptide (Figure 2A). However, the binding of HDAC1 and p66α appeared to be non-specific, as both proteins were retained by the control beads and their binding was not affected by H3K4me3. Although RbAp48 exhibited a unique H3-binding specificity that is diminished with H3K4me3, RbAp48 is unlikely to account for observed specific binding of H3 tail peptide by NURD since it is also present in the Sin3A complex that does not to bind H3 peptide (Also see Figure 3). Together these results point to CHD3 and MTA family proteins as potential candidates responsible for binding of H3 by NURD.

To test further if CHD3/4 and/or MTA family proteins mediate the binding of H3 by NURD, we resorted to a widely used benzoylphenylalanine(BPa)-based, photo-induced cross-linking technique [33,34]. BPa can form covalent bonds with proteins in a close proximity (3 Å radius) when irradiated by UV light at 360 nm. A H3 peptide with K9 replaced by a Bpa residue was synthesized. We chose to have the bulky cross-linking residue Bpa at K9 position because K9 is not the key residue for binding of NURD, but likely lies at the edge of the H3 sequence required for binding of NURD. In agreement with this idea, the H3Bpa peptide was able to bind NURD as the regular H3 peptide (Figure 2B, compare lanes 8–10 with lanes 2–4). In the absence of UV treatment, we observed that the binding of NURD to both H3 and H3Bpa peptides could be stripped from the H3 peptide by washing buffer containing 0.2% SDS (Figure 2B, compare lane 5 with lanes 2–4 and compare lane 11 with lanes 8–10). Importantly, we found that after UV treatment the binding of MTA1/2 to H3Bpa peptide but not the H3 peptide became resistant to 0.2% SDS washing (Figure 2B, compare lanes 12 and 13 with lane 11). In contrast, under the same condition the binding of CHD3 and CHD4 to the H3Bpa peptide was sensitive to 0.2% SDS washing. Similarly, the binding of HDAC1 and HDAC2, detected as a doublet in Western blot, to H3Bpa peptide was also sensitive to 0.2% SDS washing even after UV irradiation. We took this result to conclude that within the NURD complex MTA1/2 but not CHD3/4 were associated directly with the H3Bpa peptide and thus were cross-linked to the H3Bpa peptide upon UV treatment. To test if MTA3 also binds directly to H3 peptide, we carried out the same experiment with the whole cell extracts derived from Flag-MTA3 expressing HeLa cells. We observed a UV-treatment dependent, 0.2% SDS resistant association of Flag-MTA3 with the H3Bpa but not the control H3 peptide (Figure 2B). Together these data provide the first evidence that the MTA family proteins directly associate with H3 peptide and thus mediate the binding of H3 tail by NURD.

### The H3 binding activity is localized to the C-terminal regions of MTA proteins

**Figure 4:**
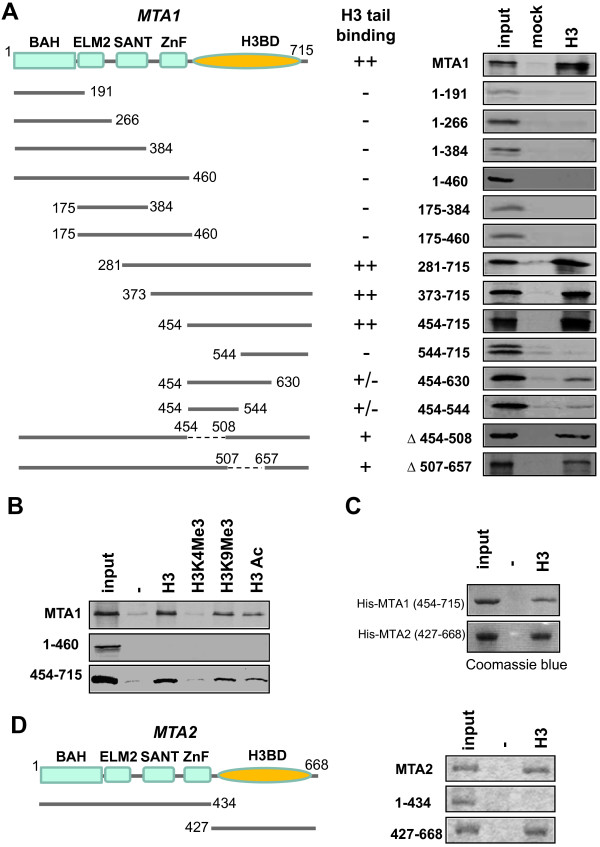
**The H3 -binding domain (H3BD) resides within the C-terminal regions of MTA proteins. A**. The binding of various MTA1 deletion mutants to H3 tail peptide in vitro. The left panel illustrates the primary structures of MTA1 and various mutants. Also indicated are four structural motifs in the N-terminal regions of MTA proteins, namely BAH, ELM2, SANT and ZnF and the C-terminal H3BD determined here. The proteins were synthesized and ^35^S-met labeled and subjected to in vitro pulldown assay with and without the immobilized H3 peptide. **B**. The C-terminal region of MTA1 exhibits the same H3 peptide-binding specificity as the full-length MTA1. The experiment was performed as in B except additional immobilized H3 peptides were included. **C**. The recombinant MTA1 and MTA2 C-terminal regions are sufficient for binding H3 tail peptide. The 6xHis-tagged MTA1(454–715) and 6xHis-MTA2(427–668) were expressed and purified from E.coli and subjected to in vitro pulldown assay with immobilized H3 tail peptide. **D**. The H3-binding activity is also mapped to the C-terminal region of MTA2. The MTA2 and its N- and C- terminal regions were expressed as ^35^S-Met labeled proteins and subjected to in vitro pulldown assay with immobilized H3 tail peptide.

To determine the region of MTA1 responsible for binding of H3 peptide, we constructed a series of MTA1 deletion mutants. These mutants were expressed as ^35^S-methionine labeled proteins by in vitro coupled transcription and translation and tested for binding to H3 tails by in vitro peptide pulldown assay. Despite the presence of SANT domain that has been implicated in binding of histones [35], we were surprised to see that all the N-terminal MTA1 constructs failed to bind H3 peptide (Figure 3A). Furthermore, the N-terminal regions including the BAH, Elm2, SANT and ZnF domains were not required for binding, as the mutants with the N-terminal deletions up to aa454 (construct 454–715) maintained the robust H3 binding activity. However, further deletion up to aa544 substantially reduced the H3 binding activity, indicating the region between aa454-544 is required for efficient binding of H3 peptide. As the aa454-544 and aa454-630 mutants displayed substantially reduced H3 binding activity, a broad region within the MTA1 C-terminus appeared to be required for H3 binding activity. Indeed, there may be more than one H3 binding module within the C-terminal region, as internal deletion of either aa454–508 or aa507-657 in the full-length MTA1 all reduced but did not abolished the H3 binding activity (Figure 4A).

To test further if the C-terminal region is responsible for binding of H3 peptide by MTA1, we also examined if the binding of MTA1(454–715) to H3 peptides was affected by H3 posttranslational modifications as the full-length MTA1. The results in Figure 3B showed that MTA1(454–715) exhibited the same binding specificity as the full length MTA1, ranging from slightly reduced binding to both acetylated and H3K9 methylated H3 peptides and substantially reduced binding to the H3K4me3 peptide.

To test if MTA1 binds directly the H3 tail peptide, we expressed and purified recombinant 6xHis-MTA1(454–715) from E.coli. Although this protein was not well expressed in E.coli, we were able to prepare enough for in vitro pulldown assay using immobilized H3 peptide. Coomassie blue staining revealed the specific binding of this recombinant protein to the immobilized H3 but not control beads (Figure 3C, upper panel). Taking together, these data allowed us to assign the H3 binding activity to the MTA1 C-terminal region that is not known for any recognizable structural and functional domain. We designated this newly identified H3-binding domain as H3BD, although the precise region of H3BD remained to be further defined.

### The MTA2 also has a C-terminal H3BD

Among the MTA family proteins, the C-terminal regions are in fact less conserved in sequences in comparison to the N-terminal regions. We next wished to determine if the the H3 binding activity of MTA2 also resides in its C-terminal domain. We tested this first by comparing the binding of in vitro translated MTA2 N-terminal fragment (1–434) and C-terminal fragment (427–668) to the H3 tail peptide in pullodwn assay. As shown in Figure 3D, the H3 binding activity was observed for the C-terminal region of MTA2 but not the N-terminal region. Furthermore, we expressed and purified the recombinant 6×His-MTA2(427–668) from E.coli. This recombinant protein was capable of binding H3 peptide as efficient as the recombinant MTA1 C-terminal domain (Figure 4C). Thus, like MTA1, the H3 binding activity of MTA2 also resides in its C-terminal domain.

### The C-terminal H3BD of MTA1 mediates the binding of H3 peptide by the core MTA1-HDAC1/2-RbAp46/48 complex

Having established that the MTA1 binds H3 tail peptide in vitro through its C-terminal H3BD, we next investigated if the H3BD of MTA1 mediates the binding of H3 peptide by NURD. For this purpose, we first expressed Flag-tagged MTA1, MTA1(1–460) and MTA1(454–715) in 293T cells and analyzed if they interacted with the endogenous HDAC1/2 and thus were incorporated into endogenous NURD complex. As shown in Figure 4A, all three proteins were expressed and could be efficiently immunoprecipitated using anti-Flag M2 beads. As expected, both HDAC1/2 and RbAp48 were efficiently co-immunoprecipitated with the full-length Flag-MTA1 (Figure 3A), indicating that the ectopically expressed Flag-MTA1 interacted with endogenous HDAC1/2 and RbAp46/48. Consistent with a previous study [28], we found CHD4 was poorly co-immunoprecipitated with the Flag-MTA1, indicating that ectopically expressed Flag-MTA1 was assembled into a core complex with HDAC1/2 and RbAp46/48 but without CHD3/4. Under the same experimental conditions, we found that MTA1 N-terminal region (F-1-460) was associated with HDAC1/2 and RbAp48, whereas the C-terminal fragment (F-454-715) was not (Figure 4A). Together these results indicate that the N-terminal 1–460 of MTA1 is sufficient for interaction and incorporation into the HDAC1/2 and RbAp46/48 core complex, whereas the C-terminal 454–715 is not incorporated into the complex.

We next analyzed the H3 tail binding property for the MTA1 proteins expressed in 293T cells. Significantly, in vitro peptide pulldown assay in Figure 4B showed that the Flag-MTA1(454–715) bound the H3 tail peptide as efficiently as full-length Flag-MTA1, whereas no binding was detected for Flag-MTA1(1–460).

To substantiate the above results further, we isolated Flag-tagged MTA1, MTA1(1–460) and MTA1(454–715) via anti-Flag immune-affinity purification as illustrated in the top panel of Figure 4C. The eluted proteins were then subjected to in vitro binding of H3 tail peptide by pulldown assay. The representative results in Figure 4C showed that the immune-affinity-purified Flag-MTA1 and its associated HDAC1/2 and RbAp48 were retained by immobilized H3 tail peptides. However, while the purified Flag-MTA1(454–715) that contained no HDAC1/2 and RbAp48 was retained by the immobilized H3 peptides, Flag-MTA1(1–460) and its associated HDAC1/2 and RbAp48 were retained poorly, if any. Taken together, these data showed that 293T-expressed Flag-MTA1(1–460) formed complex with endogenous HDAC1/2 and RbAp46/48 but exhibited a poor H3 tail-binding activity, whereas the C-terminal H3BD of MTA1 bound H3 peptide in the absence of HDAC1/2 and RbAp46/48 as efficiently as the full-length MTA1.

Unlike MTA1, ectopically expressed MTA2 is known to be assembled into the NURD complex containing HDAC1/2, RbAp46/48 and CHD3/4 - [28]. This provides us an opportunity to test the role of MTA2 C-terminal H3BD in the context of full NURD complex. We next expressed Flag-tagged full-length MTA2, the MTA2 N-terminal region 1–434 and C-terminal region 427–668 in 293T cells and analyzed their association with endogenous HDAC1/2, and CHD4 by IP-WB analysis. As shown in Figure 4D, immunoprecipitation of the full-length MTA2 and the N-terminal fragment MTA2 1–434 using anti-Flag M2 beads brought down HDAC1 as well as CHD4. However, the co-immunoprecipitation with HDAC1 and CHD4 was not observed for the Flag-tagged MTA2 C-terminal 427–668 fragment. Given that the MTA proteins are known to form exclusive NURD complexes (MTA1 or MTA2 or MTA3 does not co-exist in the same NURD complex), the co-immunoprecipitation of MTA2 1–434 fragment with CHD4 and HADC1/2 implied the incorporation of MTA2 1–434 fragment into the NURD complex by replacement of endogenous MTA proteins. When the above three 293T-expressed MTA2 proteins were subjected to in vitro peptide pulldown assay, we found that the C-terminal region free of other subunits of NURD complex was able to bind H3 tail peptide as efficient as the full-length MTA2, whereas no significant binding was observed for the N-terminal fragment that was incorporated into the endogenous NURD complex (Figure 4E). As the MTA2 1–434 fragment was incorporated into the NURD complex containing CHD3/4 presumably by replacing endogenous MTA proteins, yet no significant binding of H3 tail peptide was observed for the NURD complex containing the MTA2 1–434 fragment, this result provides a strong evidence that the MTA proteins are required for binding of H3 tail-peptide by NURD in vitro. Together these results provide compelling evidence that the MTA1 and MTA2 mediate the observed H3 tail peptide binding by NURD complex in vitro and this binding requires their C-terminal H3BD.

### The existence of H3-MTA interaction independent mechanism for NURD chromatin association

**Figure 5:**
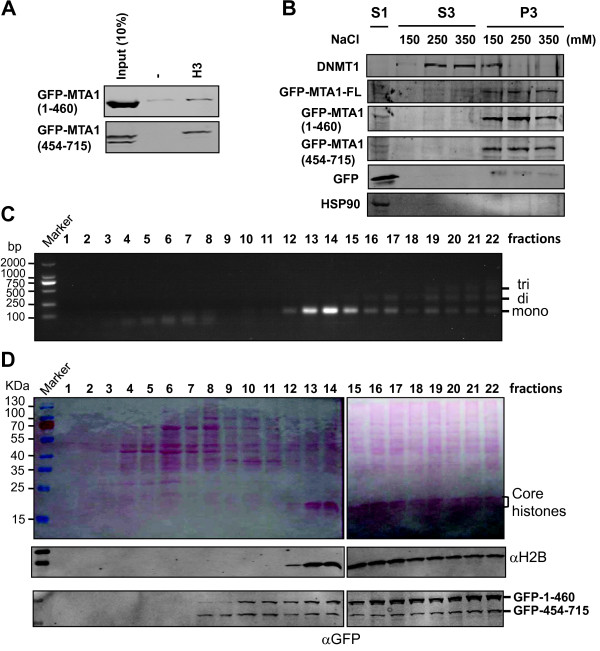
**The H3-MTA interaction independent mechanism exists for NURD chromatin association. A**. Pulldown assay using GFP-tagged MTA1 proteins confirmed the MTA1 C-terminal H3 binding activity. Note that a very weak H3 binding activity was detected for N-terminal MTA1 with intensive exposure. **B**. Both the N-terminal and C-terminal GFP-MTA1 were chromatin associated in 293T cells. The 293T cells were transfected with GFP-MTA1, GFP-MTA1(1–460) and GFP-MTA1(454–715) and the resulting cells were subjected to cellular fractionation of soluble cellular fraction and insoluble chromatin fraction as described in Materials and Methods. The fractions were further extracted with buffer containing different concentration of NaCl as indicated to generate different fractions. Note that while DNMT1 was gradually stripped from chromatin by an increasing salt concentration, all three MTA1 proteins essentially remained to be associated with chromatin. **C**. Fractionation of soluble chromatin fragments derived from MNase digestion by a 5–30% sucrose gradient centrifugation. The soluble chromatin was prepared from the 293T cells expressing both GFP-MTA1(1–460) and GFP-MTA1(454–715). After sucrose gradient centrifugation, consecutive fractions (300 μl each) were collected from top. Half of the samples were used for preparation of DNA and analyzed by agarose gel electrophoresis. **D**. The remaining half of the sucrose gradient fractionation samples was subjected to protein precipitation by TCA and resolved by SDS-PAGE. After transfer to nitrocellulose membrane, the proteins were revealed by Ponceau S staining (top panel) and then analyzed by western blot. Note that the majority of GFP-MTA1(1–460) and GFP-MTA1(454–715) were present in the chromatin fractions.

This far we have provided multiple lines of evidences that the in vitro specific binding of H3 tail peptide by NURD is likely mediated through the C-terminal H3BD of MTA family proteins. Next we wished to determine if the H3-MTA interaction is required for chromatin association of NURD complex in cells. For this purpose, we used a set of GFP-tagged MTA1 proteins that exhibited a high level of expression than the corresponding Flag-MTA1 proteins (data not shown). We first verified that like Flag-MTA1(1–460), the GFP-MTA1(1–460) expressed in 293T cells also bound poorly to the H3 tail peptide, whereas a robust binding was observed for GFP-MTA1(454–715) in a typical in vitro pulldown assay (Figure 5A). We then expressed GFP-MTA1, GFP-MTA1(1–460) and GFP-MTA1(454–715) in 293T cells by transient transfection and analyzed their chromatin association by cellular fractionation (see Materials and Methods). A representative result in Figure 5B shows that all three proteins were mainly detected in the chromatin fraction and the chromatin association was resistant to the salt extraction upon to 350 mM NaCl. Under the same experimental conditions, we found that the control GFP was dominantly presented in the cytoplasmic. Similarly, the endogenous HSP90 proteins were detected primarily in the cytoplasmic. As an internal control for chromatin fractionation, the endogenous DNMT1 was shown to be mainly chromatin associated and could be efficiently extracted from the chromatin by the buffer with 250 mM NaCl, a result consistent with a previous study [36]. These results indicate that although the MTA(1–460) fragment does not bind H3 tail peptide in vitro, it is nevertheless associated with chromatin in cells.

To further substantiate the above observation and to compare the chromatin association of MTA1(1–460) and MTA1(454–715) directly, we co-transfected both GFP-tagged MTA1 proteins into 293T cells. The cells were treated with 1% formaldehyde for 10 min to preserve the interaction of chromatin associated proteins with chromatin. We then prepared 293T cell nuclei and digested the nuclei with MNase to release chromatin into soluble fraction. The sucrose gradient (5–30%) centrifugation was then employed to separate chromatin into mononucleosomes and oligonucleosomes as judged on the basis of the sizes of DNA fragments (Figure 5C). The same fractions were then subjected to WB analysis for GFP-MTA proteins and H2B for detection of chromatin (Figure 5D). A comparison of results in Figure 5C and Figure 5D shows a nice correlation between the fractions containing nucleosomal DNA and fractions containing H2B, allowing us to identify the chromatin-containing fractions. The WB analyses using anti-GFP antibody revealed the enrichment of both GFP-MTA1(1–460) and GFP-MTA1(454–715) in the chromatin-containing fractions. No co-immunoprecipitation was observed between GFP-MTA1(1–460) and GFP-MTA1(454–715) (data not shown), thus excluding the possibility for the two MTA1 fragments to form a functional complex through complementation. Together with the salt extraction experiment, these results indicate that despite of its lack of H3 binding activity, the MTA1(1–460) fragment is associated with chromatin in cells. As the MTA1(1–460) fragment is incorporated into the HDAC1/2 and RbAp48-containing NURD complex(es), our results indicate the existence of MTA1-H3 interaction independent mechanism for association of NURD complex with chromatin.

## Discussion

### NURD is the only class I HDAC complex that binds the H3 tail with a high affinity in vitro

Accumulating evidence has demonstrated diverse roles of histone modifications in transcription and other DNA-based biological processes. In principle histone modification enzymes can be targeted to chromatin either directly through interaction with components of chromatin and/or indirectly through interaction with transcription factors or secondary regulatory proteins. Among numerous histone modification enzymes studied a recurrent theme is that the enzymes or their associated protein complexes are capable of interacting with histones [37,38]. For example, CBP and p300, two well characterized histone acetyltransferases, contain a bromodomain that recognizes acetylated lysine. The HDAC1/2-containing Sin3A corepressor complex has been shown to interact with chromatin through its associated RbAp46/48 subunits [39], whereas the NCoR/SMRT corepressor complex may interact with chromatin through the TBL1/TBLR1 subunits [40] and NCoR/SMRT [41]. However, as reported previously and confirmed in this study [19,20], the NURD complex represents the major H3 tail-binding proteins in HeLa nuclear extracts. Despite the reported RdAp46/48-histone interaction, the mSin3A complex was not observed to bind the H3 tail peptide under the same in vitro condition. Similarly, the CoREST and NCoR/SMRT complexes were not observed to bind H3 tails under the same condition (Figure 1C). Thus, the NURD complex is unique among all class I HDAC corepressor complexes tested for its specific interaction with histone H3 tail in vitro. As NURD is highly conserved in evolution and ubiquitously and abundantly expressed in cells, the specific interaction with histone H3 tail is likely to render NURD a unique role in global deacetylation of chromatin, in addition to specific targeting by various transcription factors including ikaros [42]. This idea is consistent with the widespread roles of NURD in transcription repression, DNA damage repair, cell cycle progression and genome stability [14,43-45].

### MTA family proteins as novel H3 binding proteins

A surprising finding in our study is that the MTA family proteins in the NURD complex are novel H3 binding proteins that are likely to account for specific binding of H3 tail peptide in vitro by NURD. The MTA family proteins, especially MTA1, have been shown to play important roles in diverse processes including transcriptional regulation, DNA damage repair and cancer [27,29,46-48]. Interestingly, MTA1 seemed to differ from MTA2 in association with CHD3/CHD4 as reported in a previous study [28]. The ectopically expressed MTA2 was shown to assemble into the CHD3/4-containig NURD complex, whereas ectopically expressed MTA1 was shown to form the protein complexes containing HDAC1/2 and RbAp46/48 but lacking CHD3/CHD4. In our effort to determining the protein subunit(s) responsible for H3 tail binding activity, we observed that the CHD3, MTA family proteins and RbAp48 all exhibited a H3 tail peptide binding specificity resembling to that of NURD (Figure 2A). However, our photo-induced cross-linking experiment pointed to the MTA family proteins rather than CHD3/CHD4 as the direct H3 tail peptide-binding proteins within the NURD complex (Figure 2B). Somewhat surprisingly, the H3 tail peptide binding activity was not mapped to the N-terminal regions containing SANT and zinc finger domains but to the C-terminal regions of MTA1 and MTA2 that are not known for any recognizable structural domain (Figure 3). We demonstrated that the purified recombinant C-terminal regions of MTA1 and MTA2 are capable of binding H3 tail peptide (Figure 3C), and thus designated the C-terminal region of MTA proteins as a novel H3 binding domain (H3BD) (Figure 3C). Another compelling evidence supporting the MTA proteins as the H3 tail binding protein within the NURD complex came from our analysis of 293T expressed N-terminal and C-terminal fragments of MTA1 and MTA2 (Figure 4). The N-terminal fragments of MTA1(1–460) was found to associate with endogenous HDAC1/2 and RbAp48, whereas the N-terminal MTA2(1–434) fragment was found to be incorporated into the HDAC1/2 complex containing also CHD4. However, despite being incorporated into either partial or complete endogenous NURD complex, both N-terminal fragments bound poorly the H3 tail peptide in *in vitro* pulldown assay (Figure 4B, C and E). In the contrary, although both the MTA1 C-terminal fragment 454–715 and MTA2 C-terminal fragment 427–668 were not found to associate with endogenous HDAC1/2 or CHD3/4 (Figure 4A and D), they exhibited a H3 tail peptide binding activity as efficient as the corresponding full-length MTA proteins (Figure 4B, C and E). Two conclusions can be drawn from the above results. First, as the C-terminal regions of MTA1 and MTA2 are not incorporated into the endogenous NURD complex, they are clearly the autonomous H3 tail binding domain if considering together with the H3 tail binding activity observed for their purified recombinant proteins (Figure 3C). Second, as the MTA2 N-terminal fragment 1–434 expressed in 293T cells was assembled into the NURD complex (as demonstrated by its coimmunoprecipitation with endogenous CHD4 and HDAC1 proteins) but failed to bind H3 tail peptide in vitro, the MTA proteins but not CHD3/CHD4 is required for the observed H3 tail peptide binding activity by NURD in vitro.

The MTA1 has been shown to play a role in transcriptional regulation by an increasing number of transcription factors including estrogen receptor and p53 [48,49]. It is not clear at this stage whether in all these cases MTA1 functions within and/or outside the context of NURD complex. The novel histone H3 binding activity uncovered for MTA proteins in this study provides a physical link between MTA proteins and chromatin. Future work is necessary to illustrate the structural basis for specific recognition of H3 tail by MTA family proteins.

### Multiple mechanisms are likely to target the NURD complex to chromatin

Although the MTA1(1–460) and MTA2(1–434) proteins derived from 293T cells were not detected for H3 tail peptide binding activity in vitro, we found both fragments were associated with chromatin in 293T cells (Figure 5). These results demonstrate that the NURD complex can associate with chromatin independent of the H3 binding activity of MTA proteins. One explanation is that the NURD complex can be recruited to chromatin by interaction with various transcription factors and/or other chromatin associated proteins such as KAP1/TIF1, HP1 etc. In addition, the NURD complex may associate with chromatin through the methylated DNA binding activity of its MBD3 subunit. Furthermore, although the subunits other than MTA proteins do not appear to account for in vitro H3 binding specificity of NURD, they may contribute to chromatin association in vivo either through their broad histone interaction and/or recognition of combinatorial chromatin elements. In this regard, the p66α/β subunits of the NURD complex have been shown to bind histone H3 tail as well as H2A, H2B and H4 tails [25]. In addition, both CHD3 and CHD4 subunits have been shown to bind H3 tail. The CHD3 and CHD4 proteins form exclusive NURD complexes and each contains a tandem PHD domain. The second PHD domain of CHD4 was shown to bind H3 tail peptide and more recently, the structural study revealed that the CHD4 tandem PHD domain engages in a combinatorial fashion two H3 tails in nucleosomes [23,24]. The combinatorial binding of two H3 tails by CHD4 may explain why CHD4 may not account for the H3 tail peptide binding in vitro in our experiments, yet contributes to chromatin association of NURD complex in cells. Alternatively, the specific binding of H3 tail peptide by NURD may be a combinatorial consequence of H3 tail binding by more than one subunits of the NURD complex, among them are the MTA family proteins. The presence of multiple histone binding proteins in the NURD complex may account for high affinity interaction between NURD and H3 tail and broad function of NURD in chromatin remodeling, transcription, DNA damage repair and cell cycle.

## Materials and methods

### Plasmids, peptides, antibodies and cell lines

The plasmids pCDNA3-Flag-MTA1 and pcDNA3-Flag-MTA3 were kind gift from Dr. Paul Wade (NIEHS). The plasmids for pcDNA3-CHD3, pcDNA3-MTA2, pcDNA3-p66 and pcDNA3-MDB3 were kindly provided by Dr. Yi Zhang (Univ. of North Carolina). The plasmids for HDAC1 and RbAp48and were described previously [50]. All MTA1 and MTA2 deletion mutants were generated through PCR-based cloning in the pSG5-2×Flag vector. The various H3 tail peptides used contain the H3N-terminal amino acids 1–21 followed by a GGK linker sequence and a C-terminal biotin. The H2A, H2B and H4 peptides are 1–21 aa of their corresponding N-terminal tail sequences from human core histones plus a C-terminal biotin. All peptides were synthesized and purified by Scilight Biotechnology LLC (Beijing, China). Antibodies against CHD3 and CHD4 were kindly provided by Dr. Weidong Wang (NIA). The antibodies against HDAC1/2 and NCoR were as described [40,51]. The anti-PHF8 monoclonal antibody was raised against the 6×His-PHF8(aa1-418) and as described [31]. The commercial antibodies are MTA1/2 (sc-9447, Santa Cruz), Sin3A (Santa Cruz), Flag (Sigma) and H3 (abcam). HeLa and 293T cell lines were obtained from the American Type Culture Collection (ATCC, Manassas, VA).

### In vitro pulldown of HeLa nuclear extracts or whole cell extracts with immobilized histone tail peptides

The typical peptide based pulldowns were performed essentially as described [52]. For pulldown of histone tail peptide-binding proteins from HeLa nuclear extracts followed by western blot analysis, approximately 1 μg of histone tail peptides were first immobilized onto 10 μl streptavidin-coated agarose beads and incubated with approximately 250 μg of HeLa cell nuclear extracts or whole cell extracts in 100 μl reaction with binding buffer (20 mM HEPES [pH 7.9], 150 mM KCl, 1 mM dithiothreitol [DTT], 1 mM phenylmethylsulfonyl fluoride [PMSF], 10% glycerol, 0.1% NP-40, proteinase inhibitors) for 3 h at 4°C. Unbound proteins were removed by washing the beads with washing buffer (20 mM HEPES [pH 7.9], 150 mM KCl, 1 mM DTT, 1 mM PMSF, 0.1% NP-40, proteinase inhibitors) 4 times for 5 min each. The proteins that remained bound to the peptides were separated by SDS-PAGE followed by western blotting analysis. For the experiment in Figure 1A, the pulldown reaction was scaled up to 2 mg of HeLa nuclear extract and the bound proteins were washed with the washing buffer containing 250 mM KCl.

### In-gel digestion and mass spectrometry

Sample preparation and mass spectrometry analysis on a Finnigan LTQ mass spectrometer were as described previously [53].

### In vitro pulldown of ^35^S-methionine-labeled proteins and recombinant proteins with immobilized histone tail peptides

For testing the H3 peptide-binding activity of individual subunits of NURD complex, each subunit was synthesized and ^35^S-methionine labeled using the TNT coupled reticulocyte lysate system (Promega). The reactions were diluted 1:10 with the binding buffer and subjected to binding assays as above except that the binding of the proteins was revealed by autoradiography. Similarly, various MTA1 deletion mutants were synthesized and ^35^S-methionine labeled using the TNT system and analyzed for H3 tail peptide binding by in vitro pulldown.

For testing the direct binding of H3 peptide by MTA1 and MTA2, the recombinant 6xHis-MTA1(454–715) and MTA2(427–668) were prepared from E.coli using nickel beads according to manufacturer’s instruction. The pulldown with recombinant proteins was performed essentially as above except addition of 0.5 mg/ml BSA.

### UV-induced cross-linking of H3Bpa peptide with MTA1

HeLa nuclear extracts (500 ug) or whole cell extracts derived from Flag-MTA3 expressing 293T cells were incubated with 1 ug immobilized H3 or H3Bpa peptide as described above. After extensive washing, the beads were exposed to 360 nm UV light source at a distance of ~5 cm for various times as indicated. The beads were then washed with NETN buffer (20 mM Tris pH 8.0, 200 mM NaCl, 1 mM EDTA and 1% NP40) with or without 0.2% SDS and boiled in 1xSDS loading buffer at 100°C for 30 min to reverse cross-linking. The presence of MTA family proteins and other subunits of NURD complex were examined by western blot analysis.

### Preparation of whole cell extracts and immunoprecipitation

To examine the in vitro H3-binding activity of MTA1 and its deletion mutants expressed in mammalian cells, plasmids (pSG5) encoding 2XFlag-tagged MTA1, MTA1(1–460) and MTA1(454–715) were transfected into 293T cells via lipofection. Two days after transfection the whole cell extracts were prepared and co-immunoprecipitation and pulldown with H3 peptides were performed essentially as described [52]. In addition, Flag-MTA1 and its deletion mutants were further purified from whole cell extracts using anti-Flag M2 agarose beads as described [54]. The resulting Flag-MTA1, MTA1(1–460) and MTA1(454–715) protein complexes were subjected to in vitro pulldown assay using immobilized H3 peptides.

### Chromatin isolation by cellular fractionation

To examine the chromatin association of MTA1 and its deletion mutants, plasmids (pSG5) encoding 2XFlag or GFP-tagged MTA1, MTA1(1–460) and MTA1(454–715) were transfected into 293T cells via lipofection. Two days posttransfection the cells were harvested and washed with ice-cold PBS twice. The cells were resuspended in 200 μl of buffer A (10 mM HEPES, pH7.9, 10 mM KCl, 1.5 mM MgCl2, 0.34 M sucrose, 10% glycerol, 1 mM DTT and protease inhibitor cocktail). To lyze the cells, Triton X-100 was added to a final concentration of 0.1%. The samples were incubated on ice for 8 min and centrifuged at 1500 rpm for 5 min. The resulting cytoplasmic supernatants were designated as fraction. The nuclei pellets (P1) were washed with buffer A once and the resulting nuclei pellets were lysed in buffer B (3 mM EDTA, 0.2 mM EGTA, 1 mM DTT and protease inhibitor cocktail) and incubated on ice for 30 min. The samples were centrifuged at 3500 rpm for 5 min and the resulting chromatin pellets were designated. To test the stability of MTA1 chromatin association, the pellets were resuspended with 5 volume of buffer B containing 150 mM, 250 mM and 350 mM NaCl respectively and incubated in ice for 30 min. The samples were centrifuged at 10000 rpm for 10 min and the resulting supernatants and pellets were designated. The Western blot analyses were performed using antibodies as indicated.

To examine the chromatin association by sucrose gradient centrifugation, the 293T cells expressing GFP-tagged MTA1 proteins in 10 cm culture dishes were treated with 1% formaldehyde for 10 min at room temperature with slow rotation. The cross-linking reactions were quenched by addition of glycine to 0.1 M. The cells were rinsed with PBS twice and lysed with 500 μl lysis buffer (10 mM Tris–HCl pH 7.5, 10 mM NaCl, 0.5% NP-40, 1 mM DTT and protease inhibitor cocktail). After centrifugation at 3500 rpm for 5 min to remove the supernatants, the nuclei pellets were washed with ice-cold PBS once and resuspended in 600 μl of MNase digestion buffer (50 mM Tris–HCl, pH 7.6, 1 mM CaCl2, 0.2% Triton X-100 and protease inhibitor cocktail). MNase was added to a final concentration of 0.5 U/μl and incubated at 37°C for 20 min. The digestion was stopped by addition of EDTA to a final 10 mM. The samples were centrifuged at 15000 rpm for 15 min and the resulting soluble chromatin preparations were subjected to 5-30% sucrose gradient centrifugation.

## Competing interests

The authors declare that they have no competing interests.

## Authors’ contributions

JL and JW conceived and designed the experiments. MW, LW and QL performed the experiments. MW, LW, QL, JL and JW analyzed the data. JW wrote the paper. All authors read and approved the final manuscript.
