# Correction: the MTA family proteins as novel histone H3 binding proteins

**DOI:** 10.1186/2045-3701-3-12

**Published:** 2013-02-15

**Authors:** Meng Wu, Lina Wang, Qian Li, Panagiota Karagianni, Jiwen Li, Jun Qin, Jiemin Wong

**Affiliations:** 1 Shanghai Key Laboratory of Regulatory Biology, Institute of Biomedical Sciences and School of Life Sciences, East China Normal University, 200241, Shanghai, China; 2 Biomedical Sciences Research Center Alexander Fleming, 16672, Vari, Greece; 3 Center for Molecular Center for Molecular Discovery, Verna and Marrs McLean Department of Biochemistry and Molecular Biology, Baylor College of Medicine, 77030, Houston, TX, USA

## Correction

After publication of this work [1], we noted that we inadvertently failed to include the complete list of all coauthors. The full list of authors has now been added and the Authors’ contributions and Competing interests section modified accordingly.

## Competing interests

The authors declare that they have no competing interests.

## Authors’ contributions

JL and JW conceived and designed the experiments. MW, LW, QL and PK performed the experiments. MW, LW, QL, PK, JL, JQ and JW analyzed the data. JW wrote the paper. All authors read and approved the final manuscript.
